# Returning ryegrass to continuous cropping soil improves soil nutrients and soil microbiome, producing good-quality flue-cured tobacco

**DOI:** 10.3389/fmicb.2023.1257924

**Published:** 2023-10-09

**Authors:** Hanjun Zhou, Mingjie Zhang, Jiahao Yang, Jing Wang, Yulu Chen, Xiefeng Ye

**Affiliations:** ^1^Key Laboratory of Tobacco Cultivation of Tobacco Industry, National Tobacco Cultivation & Physiology & Biochemistry Research Centre, Tobacco Science College of Henan Agricultural University, Zhengzhou, China; ^2^College of Natural Resources and Environment, Northwest A&F University, Yangling, China

**Keywords:** ryegrass, flue-cured tobacco, soil microbiome, soil fertility, quality ryegrass, quality

## Abstract

The widespread and continuous cultivation of tobacco has led to soil degradation and reduced crop yields and quality. Green manure is an essential organic fertilizer that alleviates obstacles to continuous cultivation. However, the plant–soil microecological effects of green manure on flue-cured tobacco cultivation remain unclear. Thus, a positioning trail including two treatments, chemical fertilizer application only (treatment NPK) and chemical fertilizer application with turning ryegrass (treatment NPKG) was conducted, and the effect of ryegrass returning on the soil physicochemical properties, soil microbiome, crop yield, and quality of flue-cured tobacco in continuous cropping soil were investigated. Results showed that returning ryegrass to the field increased the thickness of soil humus layer from 13 cm to 15 cm, reduced the humus layer soil bulk density to 1.29 cm^3^/g. Ryegrass tilled and returned to the field increased soil organic matter content by 6.89–7.92%, increased rhizosphere soil available phosphorus content by 2.22–17.96%, and converted the soil non-exchangeable potassium into potassium that was available for plant absorption and utilization. Ryegrass tilling and returning to the field increased the potassium content of middle leaves of flue-cured tobacco by 7.69–10.07%, the increased potassium content in flue-cured tobacco was accompanied by increased total sugar, reducing sugar, and the ratio of reducing sugar to nicotine, which facilitated the harmonization of the chemical composition of cured tobacco leaves. Moreover, the increased number of markedly improved operational taxonomic units enhanced the complexity of the soil bacterial community and its compactness after ryegrass tillage and their return to the field. The available potassium, available phosphorus, total potassium content, pH, and sampling period of the rhizosphere soil had considerable effects on the rhizosphere microbial. Ryegrass tilling and returning to the field changed the soil microbiome, which increased the abundance of bulk soil Proteobacteria, rhizosphere soil Fibrobacterota, and microbes with anti-pathogen activity (*Lysobacteria, Sphingomonas, Chaetomium, and Minimedusa*); and reduced the abundance of pathogenic fungi *Neocosmospore* genus in the soil. In brief, ryegrass returned to the field, improved soil microecology and restored soil nutrients, and established a new dynamic balance of soil ecology, thereby improving the quality of cultivated land and the quality of flue-cured tobacco.

## 1. Introduction

Flue-cured tobacco is an economically significant crop (Yang et al., 2015; Liu et al., 2020) and the main raw material for cigarette manufacturing. Its yield and quality play a crucial role in tobacco industry. Continuous cultivation is prevalent in areas ideal for tobacco cultivation, owing to factors such as fertile land resources, economic interests, and cultivation practices (Liu et al., 2020; Mirkarimi et al., 2021). Continuous cropping causes the soil to harden, the plow layer to become shallow, the organic matter content to decrease, the quality of soil fertility to decrease, and the level of land productivity to decline (Aparicio and Costa, 2007), resulting in significant economic losses and seriously affecting the sustainability of the tobacco industry.

Currently, a wide range of measures are used in field production to overcome continuous cropping challenges, including selecting appropriate cropping methods, such as rotation, intercropping, and cover crops (Yang et al., 2017; Li and Wu, 2018; Esmaeilzadeh-Salestani et al., 2021; Gao et al., 2022), proper soil fertilization, such as green manure (Huang et al., 2019; He et al., 2020; Chamkhi et al., 2022), and biological control (Liu et al., 2018). Green manure has been traditionally used since China’s long history of agricultural production (Wang T. et al., 2022). De Santos et al. (2018) found that Melons intercropped with green manure not only produce cleaner fruit but also enhance the environment and economy. Yang et al. (2022) reported that replacing urea-N with green manure (*Astragalus sinicus* L.) mitigates CH_4_ and NO_2_ emissions in rice paddy. Green manure has attracted the interest of many researchers due to its ability to accomplish both food safety and green farming development (Kumar et al., 2011; Liang et al., 2022).

Soil microbiome play a crucial role in soil nutrient conversion, cycling, and maintaining soil ecological functions and health. Alterations in the microbial community are one of the main obstacles to continuous cropping in tobacco production (Chen et al., 2022). Green manure crops are organic fertilizers (Wang X. Y. et al., 2022) that can influence soil microbiome directly through the nutrients in the green manure itself, or indirectly through changes in soil and plant characteristics. Currently, researches on soil microbiology in tobacco field is still under continuous cropping conditions (Wang M. et al., 2022). Few studies have been conducted on the effects of green manure on soil characteristics, soil microbiome, and flue-cured tobacco yield and quality.

The Henan flue-cured tobacco growing region, which has a more than 100-year history of cultivation, is representative of China’s strong-flavored flue-cured tobacco. The soil types in the Henan flue-cured tobacco growing region are mostly loamy, which is well aerated and has good water and fertilizer retention capacity. In recent years, the shortage of land resources has led to a severe succession of crops, resulting in soil degradation and poor nutrient availability for flue-cured tobacco plants. Therefore, it is essential to enhance the soil quality in the Henan flue-cured tobacco growing region in order to improve the yield of flue-cured tobacco. Ryegrass is an important source of green manure. In this study, a positioning trail including two treatments, chemical fertilizer application only (treatment NPK) and chemical fertilizer application with turning ryegrass (treatment NPKG) was conducted, and the effect of ryegrass returning on the growth and development of flue-cured tobacco, soil properties, and soil microbiome in continuous cropping soil were investigated. Further, the relationship between soil physicochemical properties and soil microbiome was analyzed. This research provides fundamental knowledge that mitigates the detrimental effects of continuous cultivation and enhances the quality and yield of flue-cured tobacco to promote sustainable agricultural production.

## 2. Materials and methods

### 2.1. Study area and experimental design

The investigation was conducted in the Science and Education Park of Henan Agricultural University (113°35′51″E, 34°52′13″N), Zhengzhou, China. It has a north temperate continental monsoon climate, with an average annual temperature of 14°C, an average annual rainfall of about 640 mm, a frost-free period of 220 days, and 2,400 h of annual sunlight. Ryegrass was planted for three seasons before the start of the positioning trial to ensure homogeneous soil fertility. The first ryegrass was sown on April 28, 2017, and was removed from the field on September 5, 2017. On September 22, 2017, the ryegrass was sown for the second time, and on May 10, it was removed from the field. Ryegrass was sown for the third time on May 26, and removed from the field on August 31, 2018. After homogenizing the fields, ryegrass was planted on October 27, 2018, and turned over on March 28, 2019. We started growing flue-cured tobacco in 2019 and returned the entire amount of ryegrass to the tilling plots planted with ryegrass. Before tilling, two 1 m^2^ plots representing the overall growth of ryegrass in the plot were selected. The ryegrass was dug out with the roots, and the amount of tillage in each plot was counted after removing the soil and impurities. The ryegrass tilled in 2019, 2020, and 2021 were 6297.58, 7807.68, and 7496.53 kg/hm^2^ (on dry a weight basis), respectively. Ryegrass was irrigated, tilled, and not fertilized during the planting period. The test soil was sandy loam with the following basic physicochemical properties: pH 7.57, organic matter (OM) 16.76 g/kg, total nitrogen (TN) 0.69 g/kg, alkaline nitrogen (AN) 32.40 mg/kg, available phosphorus (AP) 29.19 mg/kg, and available potassium (AK) 135.47 mg/kg.

Two treatments were tested: no ryegrass, chemical fertilizer application only (treatment NPK) and chemical fertilizer application with turning ryegrass (treatment NPKG). Each treatment was replicated three times for six plots, and each plot with an area of 66 m^2^ (12 m × 5.5 m) was arranged in randomized groups. Cement slabs (100 × 50 × 4 cm, 80 cm buried in the soil) were set around the plot to prevent fertilizer and water from crossing the irrigation. The dosage of N was 52.5 kg/hm^2^, N: P_2_O_5_: K_2_O were 1: 2: 3. Special compound fertilizers (N: P_2_O_5_: K_2_O = 8: 12: 20), potassium sulfate (K_2_O 52%), and calcium magnesium phosphate (P_2_O_5_ 16%) were used as base fertilizers, which were applied by band application before transplanting. Potassium nitrate (N: P_2_O_5_: K_2_O = 13.5: 0: 44.5) was applied as topdressing and dissolved in water for hole application.

The tobacco variety tested was Zhongyan 100, with 110 cm row spacing and 50 cm plant spacing. Seedling transplantation was performed on April 30, 2020, and April 29, 2021, and harvesting was completed on September 15, 2020, and September 6, 2021. Other management measures were implemented in accordance with local management standards for quality tobacco leaves.

### 2.2. Sample collection and measurements

#### 2.2.1. Soil sample collection

Bulk and rhizosphere soils were collected 45 and 75 days after transplanting the tobacco seedlings, respectively. Bulk soil collection: The middle position of two tobacco plants was collected at 0–20 cm using a soil auger. Five soil samples were collected from each plot and mixed as one sample. Rhizosphere soil collection: Sampling was carried out using destructive methods. The complete tobacco root system was dug and tapped to allow the loose soil combined with the roots to fall naturally. Then, the soil that was tightly combined with the root system (within 2 mm) was placed into the sampling bag, and the rhizosphere soil samples were collected. Three tobacco plants were selected from each plot to collect the rhizosphere soil, and the rhizosphere soil from the same treatment was mixed as one sample. Each soil sample was divided into two parts: one part was stored in a refrigerator at −80°C for high-throughput sequencing of soil microbrome, and the other was dried in the shade and screened for soil physicochemical indicators. After the flue-cured tobacco was mature and harvested, the soil profile was observed, and soil samples from different soil layers were collected to determine their bulk density.

#### 2.2.2. Determination of physicochemical properties of soil

The ring-knife method was used to determine bulk soil density and soil pH was determined using a pH meter (soil: water = 1: 2.5) (Bao, 2000). AN and TN were determined using the diffusion and Kjeldahl methods, respectively; AP and total phosphorus (TP) were determined using the molybdenum antimony sulfate anti-colorimetric method and NaOH alkali fusion-molybdenum antimony anti-spectrophotometric method, respectively (Bao, 2000); and AK and total potassium (TK) were determined using the flame photometric method and the standards of NY/T87-1988 standards, respectively.

#### 2.2.3. Flue-cured tobacco sample collection and determination

Before harvesting the flue-cured tobacco in 2020 and 2021, three representative tobacco plants were selected for each plot. The height, stem circumference, maximum leaf length, and maximum leaf width of each tobacco plant were determined according to the standard method of YC/T 142–2010. The selected tobacco plant was then centered on the stem base, and the surrounding 30 cm root belt soil was removed. The roots, stems, and leaves of the tobacco were separated and washed with tap water and deionized water. The tobacco plant was placed in an oven for 15 min at 105°C and then dried at 65°C until constant weight.

A continuous flow analyzer (AutoAnalyzer 3, Brown Ruby, Germany) was used to determine the total sugar, reducing sugar, nicotine, chlorine, and potassium contents of flue-cured tobacco leaves according to the standards YC/T159-2002, YC/T160-2002, YC/T217-2007, and YC/T162-2011, the ratio of potassium to chloride, the ratio of reducing sugar to nicotine, and the ratio of reducing sugar to total sugar.

#### 2.2.4. DNA extraction, PCR amplification, and high-throughput sequencing

DNA was extracted from the soil samples using the MagPure Soil DNA KF Kit. Subsequently, the purity and concentration of DNA were detected by agarose gel electrophoresis, and PCR amplification was performed using specific primers with barcodes and high-efficiency, high-quality fidelity enzymes. Bacterial amplification was performed using 343F 5’-TACGGRAGGCAGCAG-3′ and 798R 5’-AGGGTATCTAATCCT-3′. The internal transcribed spacer (ITS) segments of fungal amplification, ITS1F 5’-CTTGGtcatttagaggaagtaa-3′ and ITS2 5’-GCTGCGTTCTTCATCGATGC-3′. Shanghai Ouyi Biomedical Technology was commissioned to perform the sequencing and biological information analysis using a MiSeq sequencer (Illumina). The raw tags were trimmed, merged, and assigned using the split library software (version 1.8.0) in QIIME to remove sequences with more than eight single-base repeats and sequences with lengths less than 200 bp to obtain clean tag sequences. The quality sequence valid tags obtained from the quality control were classified into operational taxonomic units (OTUs) based on 97% similarity using the Vsearch (version 2.4.2) software, and the most abundant sequence in each OTU was selected as the typical sequence of that OTU.

### 2.3. Statistical analysis

The least significant difference (LSD) test in one-way analysis of variance was used to analyze the differences and significance of soil physicochemical properties, flue-cured tobacco agronomic traits, dry matter accumulation, and soil microbial diversity among the different treatments. Co-occurrence network analysis was performed on OTUs with a total abundance in the top 200 to characterize the complex links between microbial communities. OTUs that were statistically significantly correlated were calculated using the “psych” package in R (Spearman coefficient ρ ≥ 0.7, *p* < 0.05). The microbial co-occurrence networks for the different treatments were graphed using Gephi software (version 0.9.2), and the topological properties of the networks were calculated. Principal component analysis (PCoA) based on the Bray-Curtis distance matrix was used to analyze the differences in the beta diversity of the microbial community. Redundancy analysis (RDA) was performed using the “vegan” package in R (4.2.2) to assess the relationship between soil physicochemical factors and soil microbial communities. Linear discriminant analysis effect size (LEfSe) was used to search for biomarkers that differed significantly between treatments, with a linear discriminant analysis score of two.

## 3. Results

### 3.1. Soil forming layer profile and soil bulk density

**Figure 1:**
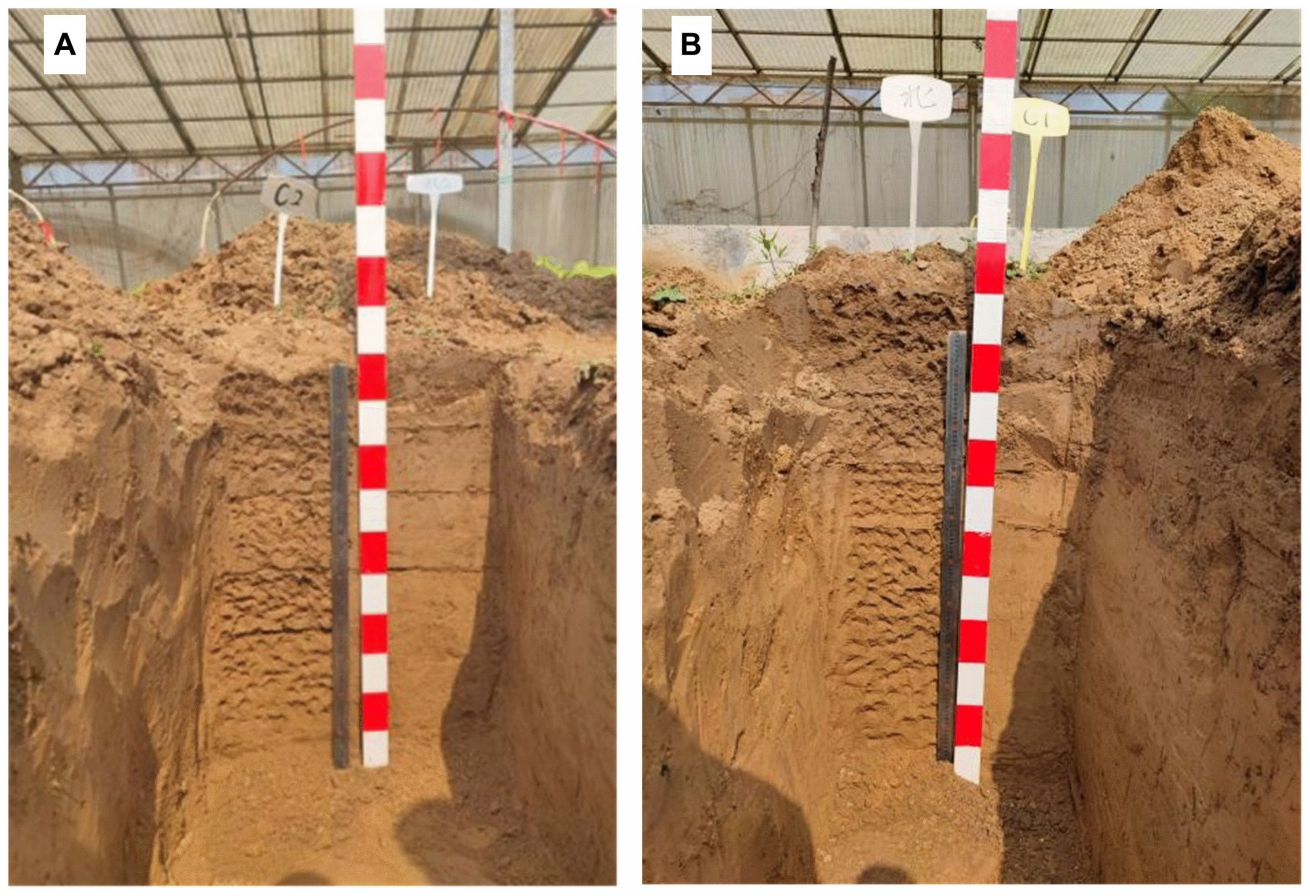
Soil profiles of **(A)** NPK and **(B)** NPKG. NPK, no ryegrass, chemical fertilizer application only; NPKG, chemical fertilizer application with turning ryegrass.

The profile of the soil-forming layer and thicknesses of different soil-forming layers are shown in Figure 1 and Table 1, respectively. As shown in Figure 1, the soil profile of the NPKG treatment differed from that of the NPK treatment. Compared to the NPK treatment, the soil texture of the NPKG humus layer was looser. The deposition layer contained coarser particles and had higher soil compactness than the humus layer. The soil deposition layer of the NPKG treatment was more compact and contained more clay particles, but the soil particles were coarser than those of the NPK treatment. Table 1 shows that the thicknesses of the humus, leaching, deposition, and parent material layers in the NPKG treatment were 0–15 cm, 15–30 cm, 30–50 cm, and below 50 cm, respectively. The thicknesses of the humus, leaching, deposition, and parent material layers in the NPK treatment were 0–13 cm, 13–30 cm, 30–45 cm, and < 45 cm, respectively. The thicknesses of the humus and deposition layers in the NPKG treatment were greater than those in the NPK treatment. Soil bulk density of the humus, leaching, and deposition layers showed increasing trends (Table 1). The soil bulk density of the humus and leaching layers of NPKG was significantly lower than that of NPK.

**Table 1:** The thickness and soil bulk density of different soil generating layer.

Soil generating layers	Soil generating layer thickness (cm)		Soil bulk density (cm^3^/g)	
	NPK	NPKG	NPK	NPKG
Humus layer	0–13	0–15	1.33 + 0.010a	1.29 + 0.004b
Leaching layer	13–30	15–30	1.38 + 0.020a	1.33 + 0.016b
Deposition layer	30–45	30–50	1.44 + 0.050a	1.48 + 0.007a
Parent material layer	less than 45	less than 50	1.39 + 0.029a	1.30 + 0.024a

Different lowercase letters indicate significant difference between treatments NPK and NPKG (*P* < 0.05). NPK, no ryegrass, chemical fertilizer application only; NPKG, chemical fertilizer application with turning ryegrass.

### 3.2. Soil fertility

Soil fertility is critical to crop yield and quality; it can be seen from Table 2 that tilling ryegrass in 2020 had no significant effect on either rhizosphere or bulk soil pH, while the pH of both rhizosphere and bulk soil decreased after turning and pressing ryegrass back into the field in 2021. At 45 days after transplanting in 2021, the bulk soil pH of the treatment NPKG was significantly lower than that of the treatment NPK. The soil AN content was 27.18–31.03 mg/kg for treatment NPK and 29.58–33.52 mg/kg for treatment NPKG between 2020 and 2021, where the AN content in rhizosphere soil was 11% higher for treatment NPKG than treatment NPK at 45 days after the transplant in 2020. The AN content of bulk soil was relatively high at 45 days after transplanting in 2020 and 2021, which showed a decreasing trend with the extension of the growth period. The soil AP content also decreased gradually as the growth period of the tobacco plants was prolonged. The AP content in bulk soil was 46.08–58.80 mg/kg in 2020 and 41.17–63.86 mg/kg in 2021, respectively. The AP content in rhizosphere soil was 56.46–66.60 mg/kg in 2020 and 56.67–74.90 mg/kg in 2021, respectively. The AP content was higher in the rhizosphere than in the bulk soil during each period, and rhizosphere soil AP content increased by 2.22–17.96%. As shown in Table 2, 45 days after transplanting in 2020 and 2021, the bulk and rhizosphere soil AK contents of the NPKG treatment were significantly higher than those of the NPK treatment. The soil AK content of treatment NPKG was 230.33–267.33 mg/kg and 270.67–380.80 mg/kg in 2020 and 2021, respectively. The results also indicated that the soil AK content increased with the number of years of ryegrass tillage. Soil OM content of treatment NPK and NPKG was 14.65–15.96 g/kg and 15.81–17.06 g/kg, respectively, and TN content was 0.81–0.90 g/kg and 0.85–0.98 g/kg, respectively (Table 2). The OM content of NPKG was 6.89–7.92% times higher than treatment NPK. There were no significant differences in soil TN and TP contents between treatments in most periods, except for significant differences between treatments in individual periods. There was also no significant difference in the soil TK content between the treatments. These results indicate that tilling ryegrass is beneficial for increasing the soil bulk and rapidly available rhizosphere nutrients, especially soil AK and rhizosphere soil AP content.

**Table 2:** Physicochemical properties of NPK and NPKG under different stages.

Stage		Treatment	pH	AN (mg/kg)	AP (mg/kg)	AK (mg/kg)	TN (g/kg)	TP (g/kg)	TK (g/kg)	OM (g/kg)
2020-45d	Bulk soil	NPK	7.67±0.13 a	28.93±1.07 b	53.84±3.86 b	175.00±12.29 c	0.86±0.02 a	1.10±0.00 c	17.09±0.13 a	15.62±0.41 b
		NPKG	7.70±0.06 a	32.90±1.40 a	58.80±5.28 ab	230.33±8.15 ab	0.93±0.04 a	1.13±0.01 bc	17.09±0.03 a	16.66±0.36 a
	Rhizosphere soil	NPK	7.72±0.03 a	28.31±1.17 b	63.86±3.97 ab	210.33±16.04 b	0.87±0.03 a	1.18±0.03 ab	17.30±0.15 a	15.70±0.36 b
		NPKG	7.60±0.02 a	31.43±1.42 ab	65.28±3.56 a	249.33±1.53 a	0.93±0.04 a	1.19±0.02 a	17.28±0.19 a	17.06±0.27 a
2020-75d	Bulk soil	NPK	7.89±0.09 a	27.88±1.99 a	46.08±0.77 c	194.00±24.27 b	0.84±0.03 a	1.12±0.03 b	16.73±0.17 a	15.89±0.48 a
		NPKG	7.88±0.05 a	30.68±1.65 a	50.76±2.48 bc	237.33±5.03 a	0.88±0.07 a	1.16±0.01 b	17.14±0.16 a	16.66±0.53 a
	Rhizosphere soil	NPK	7.74±0.07 ab	27.18±2.47 a	56.46±4.77 b	236.33±7.02 a	0.83±0.02 a	1.15±0.08 b	16.66±0.25 a	15.96±0.62 a
		NPKG	7.65±0.02 b	29.68±1.40 a	66.60±1.85 a	267.33±11.06 a	0.90±0.05 a	1.29±0.00 a	17.39±0.51 a	16.77±0.54 a
2021-45d	Bulk soil	NPK	7.80±0.04 a	31.03±0.42 b	54.74±1.69 c	254.33±23.50 b	0.90±0.02 ab	1.05±0.04 a	18.08±0.12 a	15.48±0.48 ab
		NPKG	7.60±0.12 b	33.52±0.64 a	63.86±5.68 bc	380.80±24.12 a	0.98±0.06 a	1.05±0.13 a	18.41±0.22 a	16.09±0.77 a
	Rhizosphere soil	NPK	7.88±0.06 a	28.61±1.11 c	67.30±3.52 ab	267.60±11.66 b	0.86±0.02 b	1.06±0.05 a	18.15±0.20 a	14.65±0.23 b
		NPKG	7.70±0.05 ab	29.82±0.73 bc	74.90±2.02 a	350.40±15.95 a	0.93±0.06 ab	1.04±0.09 a	18.08±0.40 a	15.81±0.47 a
2021-75d	Bulk soil	NPK	7.93±0.03 a	27.61±0.73 a	41.17±1.78 d	241.33±17.93 ab	0.85±0.02 a	1.22±0.12 a	17.74±0.10 a	15.30±0.42 b
		NPKG	7.86±0.03 a	29.82±1.92 a	47.85±2.02 c	299.33±11.37 a	0.88±0.03 a	1.10±0.09 a	17.85±0.21 a	16.58±0.24 a
	Rhizosphere soil	NPK	7.90±0.05 a	28.37±0.64 a	56.67±1.98 b	237.33±11.55 b	0.93±0.06 a	1.13±0.11 a	18.16±0.18 a	15.30±0.35 b
		NPKG	7.83±0.10 a	29.58±1.51 a	62.14±1.98 a	270.67±40.46 ab	0.90±0.04 a	1.09±0.07 a	17.85±0.22 a	16.19±0.86 ab

Different lowercase letters indicate the significant difference of soil physicochemical properties between treatments NPK and NPKG (*P* < 0.05). AN, alkaline nitrogen; AP, available phosphorus; AK, available potassium; TN, total nitrogen; TP, total phosphorus; TK, total potassium; OM, organic matter. NPK, no ryegrass, chemical fertilizer application only; NPKG, chemical fertilizer application with turning ryegrass.

### 3.3. Agronomic traits, dry matter accumulation, and chemical constituents of flue-cured tobacco

**Figure 2:**
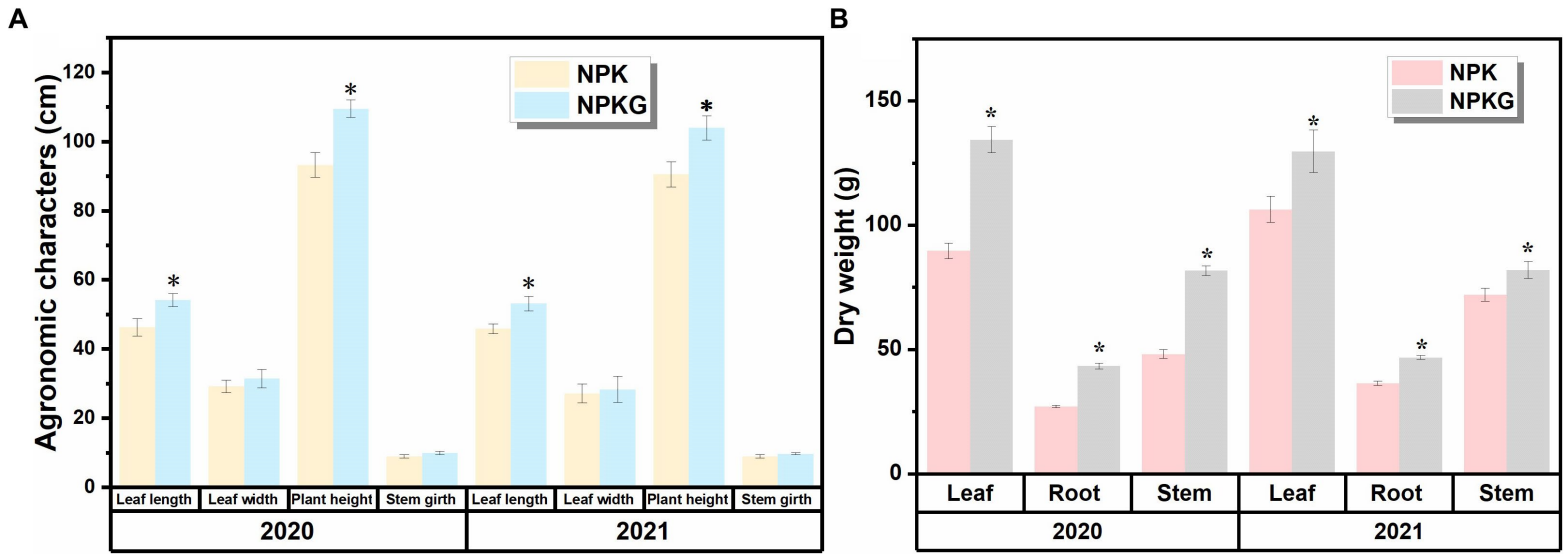
Flue-cured tobacco agronomic traits and dry matter accumulation of treatments **(A)** NPK and **(B)** NPKG. * represents *p* < 0.05. NPK, no ryegrass, chemical fertilizer application only; NPKG, chemical fertilizer application with turning ryegrass.

As shown in Figure 2, all agronomic traits in the NPKG treatment were better than those in the NPK treatment. The dry leaf weight in treatments NPK and NPKG were 89.7–106.4 g and 129.8–134.4 g, respectively. The root, stem, and leaf dry weights of NPKG in 2021 increased by 21.93, 13.80, and 28.45%, respectively, compared with those of the NPK treatment.

The chemical composition of the flue-cured tobacco leaves is shown in Table 3. The chlorine and nicotine contents of the leaves were 0.67–0.83% and 2.63–3.23%, respectively. Compared with the NPK treatment, NPKG reduced the nicotine content in the middle and upper leaves and increased the chloride content, but neither of the two treatments showed statistically significant differences in either indicator. The total sugar contents of the upper and middle leaves in the NPK treatment were 18.33–18.93% and 18.20–18.77%, respectively, whereas the total sugar contents of the upper and middle leaves in the NPKG treatment were 20.66–24.36% and 20.49–21.37%, respectively. The reducing sugar contents of NPK and NPKG were 13.37–14.94% and 15.09–16.20%, respectively. The reducing sugar to nicotine ratio of flue-cured tobacco was 4.34–4.71 and 5.14–5.73 for treatments NPK and NPKG, respectively. The reducing sugar content and the ratio of reducing sugar to nicotine in the NPKG treatment were significantly higher than those in the NPK treatment. The potassium content of the upper and middle leaves in the NPKG treatment increased by 3–10% compared to that in the NPK treatment. Ryegrass ripping and returning to the field increased the total sugar, reducing sugar and potassium content, and the reducing sugar-to-nicotine ratio in the upper and middle leaves, indicating that ryegrass turning and returning to the field is conducive to improving the quality of flue-cured tobacco leaves.

**Table 3:** Chemical composition of flue-cured tobacco.

			Chloride %	Nicotine %	Reducing sugar %	Total sugar %	Potassium %	Ratio of potassium to chloride	Ratio of reducing sugar to nicotine	Ratio of reducing sugar to total sugar
2020	Middle leaves	NPK	0.75a	3.16a	14.94b	18.77a	1.39b	1.88a	4.76b	0.64a
		NPKG	0.82a	2.88a	15.46a	21.37a	1.53a	1.87a	5.37a	0.68a
	Upper leaves	NPK	0.78a	3.23a	14.67b	18.93b	1.30b	1.68a	4.44b	0.75a
		NPKG	0.83a	3.10a	16.20a	24.36a	1.38a	1.67a	5.23a	0.66a
2021	Middle leaves	NPK	0.74a	2.84a	13.37b	18.20b	1.43b	1.94a	4.71b	0.73a
		NPKG	0.78a	2.63a	15.09a	20.49a	1.54a	1.98a	5.73a	0.74a
	Upper leaves	NPK	0.67a	3.17a	13.75b	18.33b	1.32a	1.95a	4.34b	0.75a
		NPKG	0.70a	3.09a	15.90a	20.66a	1.36a	1.93a	5.14a	0.77a

Different lowercase letters indicate the significant difference of flue-cured tobacco chemical composition between treatments NPK and NPKG (*p* < 0.05). NPK, no ryegrass, chemical fertilizer application only; NPKG, chemical fertilizer application with turning ryegrass.

### 3.4. Analysis of soil microbial community diversity and co-occurrence network

The bacterial 16S rRNA gene V3–V4 region was sequenced using the Illumina HiSeq high-throughput sequencing platform, the tags were cleaned up after removing the chimeras, and the number of valid tags (valid sequences) data was obtained with a distribution of 65,558–72,622. The fungal ITS rRNA was sequenced, and the data volume of valid tags obtained by removing the chimeras of clean tags was distributed in 38,028–64,929 (Supplementary Table S1). There was no significant difference in the number of soil bacterial and fungal OTUs between the NPKG and NPK treatments. As shown in Supplementary Table S1, the coverage of each sample was above 96%, indicating that the sequencing depth reflects the actual microbial community. As shown in Supplementary Table S2, the Chao1 index of bulk and rhizosphere soil bacteria was notably higher in 2021 than in 2020; however, the Chao1 index of bulk soil fungi showed the opposite trend. The Chao1 index of bulk and rhizosphere soil fungi and bacteria tended to be higher than that of the NPK treatment at all periods after ryegrass tillage and return to the field.

**Figure 3:**
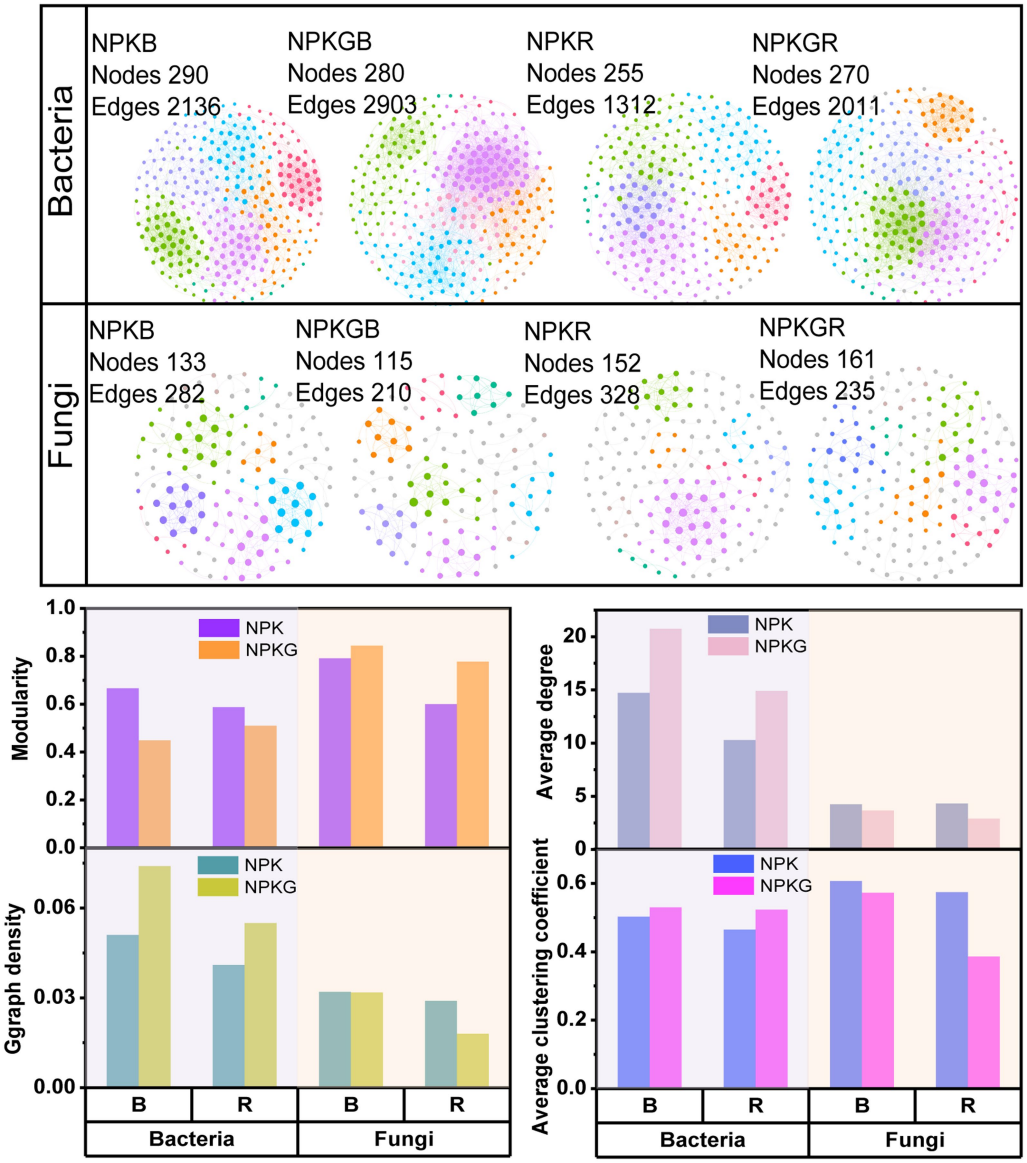
The co-occurrence networks and topological properties of bulk and rhizosphere soil bacteria and fungi between different treatments. Different colors represent different modules in the microbial networks. NPK, no ryegrass, chemical fertilizer application only; NPKG, chemical fertilizer application with turning ryegrass. B and R represent bulk soil and rhizosphere soil, respectively. NPKB, and NPKGB represent the bulk soil of the treatment NPK and NPKG, respectively; NPKR, and NPKGR represent the rhizosphere soil of the treatment NPK and NPKG, respectively.

Co-occurrence network analysis of the soil microbial community was conducted at the OTU level to further explore the complexity of the microbial community relationships among the different treatments. As shown in Figure 3, the numbers of soil bacterial edges for treatments NPKGB and NPKGR were 2,903 and 2011, respectively, which were 1.36 and 1.53 times higher than those for NPKB and NPKR, respectively. The average degree, graph density, and average clustering coefficient of the NPKGB and NPKGR treatments were higher than those of the NPKB and NPKR treatments, indicating that ryegrass tilling and returning to the field increased the interaction complexity of the soil bacterial co-occurrence network. The topological properties of the soil fungal co-occurrence network community after ryegrass rolling showed a trend opposite to that of the soil bacterial community. The number of edges and average clustering coefficient of the fungal co-occurrence network of bulk and rhizosphere soils with ryegrass in the field were lower than those without ryegrass. The results demonstrated that the soil bacterial community had higher complexity and stronger compactness after ryegrass tilling and its return to the field.

**Figure 4:**
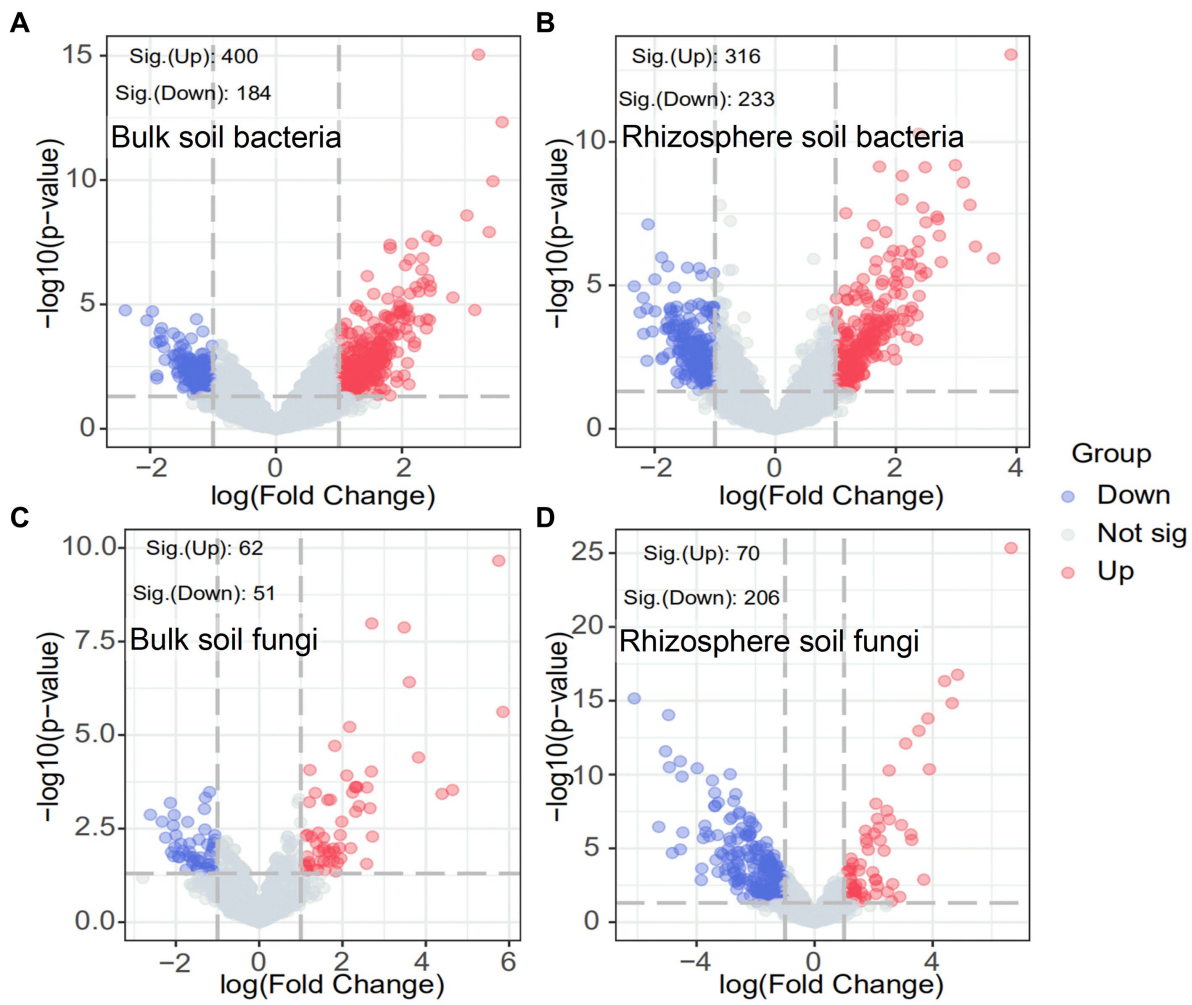
Volcano plots of bulk and rhizosphere soil bacteria **(A,B)** and fungi **(C,D)**. Blue circles indicate significantly decreased OTUs, red circles indicate significantly increased OTUs and gray circles indicate OTUs with no significant change.

The response of the soil microbial community after ryegrass tillage and return to the field at the OTU level was specifically analyzed using volcano plots. As shown in Figure 4, the number of significantly increased OTUs in both bulk and rhizosphere soils substantially improved after ryegrass was turned and pressed into the field: 400 OTUs significantly increased and 184 OTUs significantly decreased in bulk soil bacteria, 316 OTUs significantly increased, and 233 OTUs significantly decreased in rhizosphere soil bacteria. In contrast, the number of significantly decreased OTUs was greater than that of significantly increased OTUs in the bulk soil fungi. A total of 206 OTUs significantly decreased, and 70 OTUs significantly increased after ryegrass tillage and return to the field. The results demonstrated the contribution of ryegrass to the increase in the soil bacterial community.

**Figure 5:**
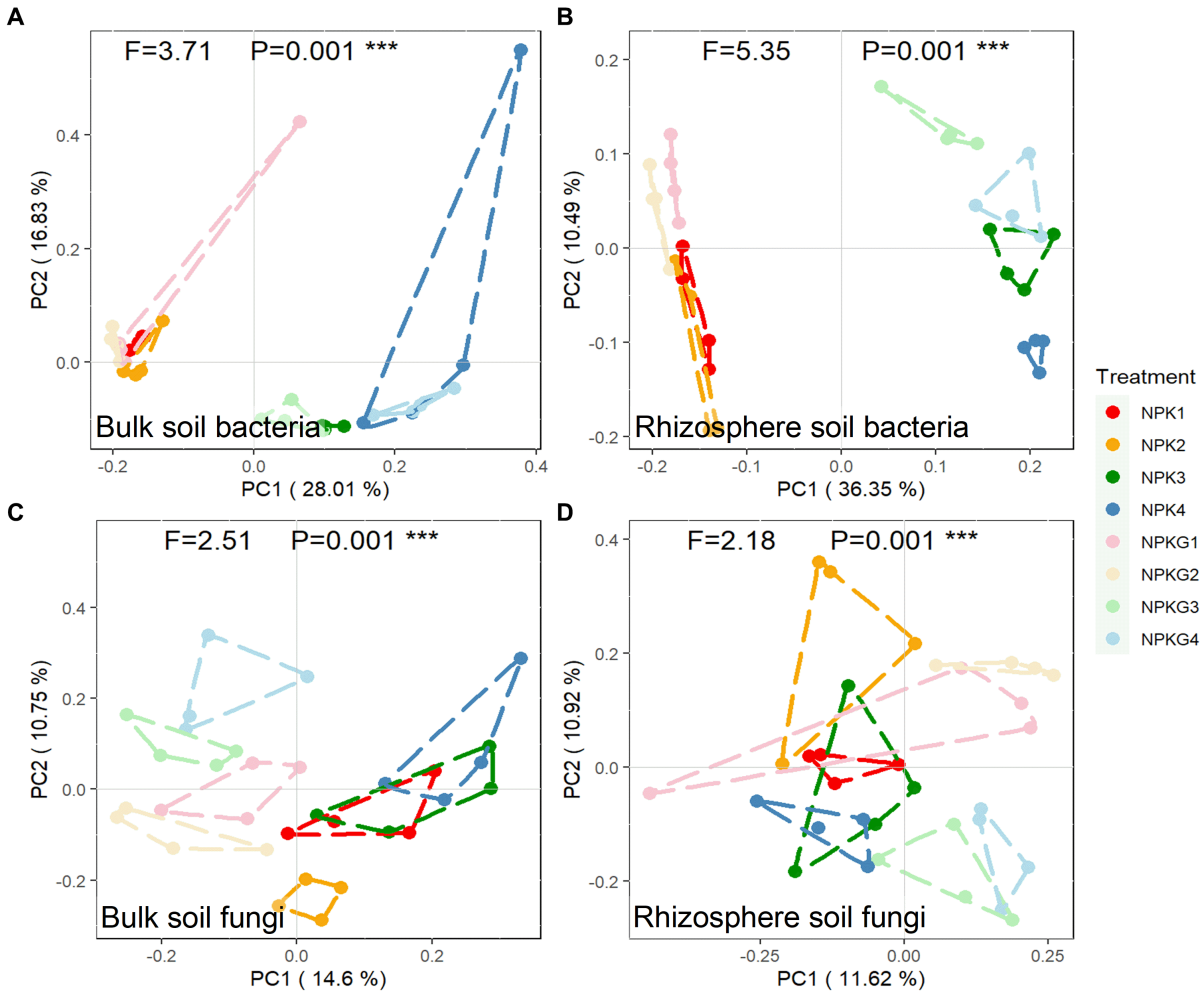
PCoA of **(A)** bulk soil bacteria, **(B)** rhizosphere soil bacteria, **(C)** bulk soil fungi, and **(D)** rhizosphere soil fungi community composition at OTU level (*n* = 4). NPK, no ryegrass, chemical fertilizer application only; NPKG, chemical fertilizer application with turning ryegrass. 1, 2, 3, and 4 in treatments represent 45 days and 75 days after transplanting in 2020, respectively, and 45 days and 75 days after transplanting in 2021.

To further clarify the differences in the microbial community composition among the samples, the soil bacterial and fungal communities were analyzed by PCoA based on the Bray-Curtis algorithm, as shown in Figure 5. For both bulk and rhizosphere soil bacteria (Figures 5A,B), samples from the same year were clustered together, and samples from different treatments in 2021 were dispersed from each other. Treatments NPK1, NPK2, NPKG1, and NPKG2 clustered together, while there was no aggregation between treatments NPK1 and NPK3, indicating that the bulk and rhizosphere soil bacterial communities were highly variable between years and that tilling and returning ryegrass caused variation in the bulk soil bacterial community. For the PCoA analysis of bulk soil fungi, NPK1, NPK3, and NPK4 were combined. Treatments NPKG1, NPKG2, NPKG3, and NPKG4 tended to aggregate, indicating that returning ryegrass to the field changed the bulk soil fungal community.

**Figure 6:**
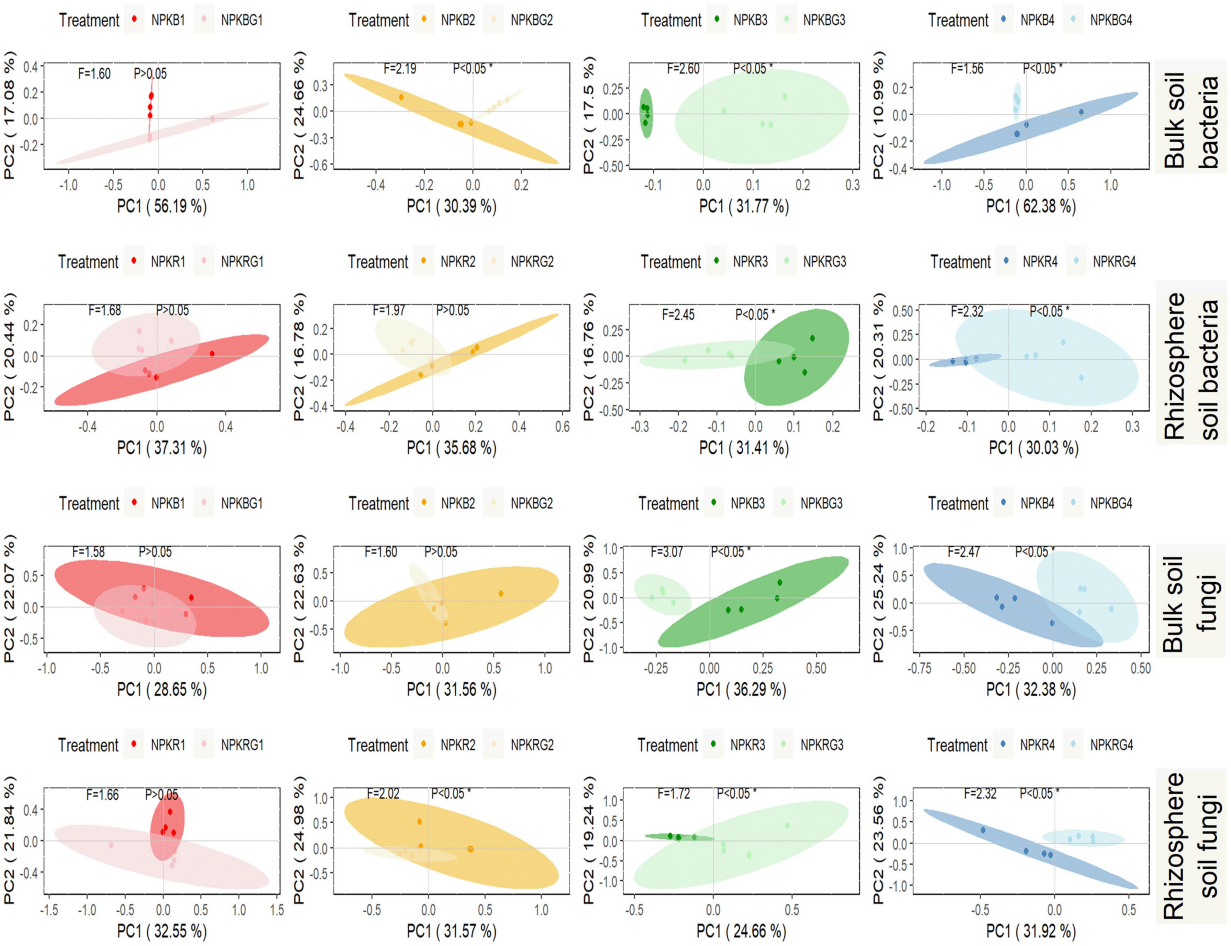
PCoA of bulk soil bacteria, rhizosphere soil bacteria, bulk soil fungi, and rhizosphere soil fungi community composition at OTU level. Ellipses are calculated with a confidence of 0.05. NPKB, and NPKBG represent the bulk soil of the treatment NPK and NPKG, respectively; NPKR, and NPKRG represent the rhizosphere soil of the treatment NPK and NPKG, respectively. 1, 2, 3, and 4 in treatments represent 45 days and 75 days after transplanting in 2020, respectively, and 45 days and 75 days after transplanting in 2021.

PCoA was performed on the soil microbial communities of the NPK and NPKG treatments for the same period to further explore the timing effect on the soil microbial community after ryegrass tillage and return. As shown in Figure 6, there was no significant difference in the bacterial and fungal communities in the bulk and rhizosphere soils between the NPK and NPKG treatments during the first sampling period. The bulk and rhizosphere soil bacterial and fungal communities in the third and fourth sampling periods showed significant differences between the NPK and NPKG treatments (*p* < 0.05). The results indicated that ryegrass tilling and returning to the field for 3 years significantly changed the soil microbiome.

### 3.5. Analysis of soil microbiome composition

**Figure 7:**
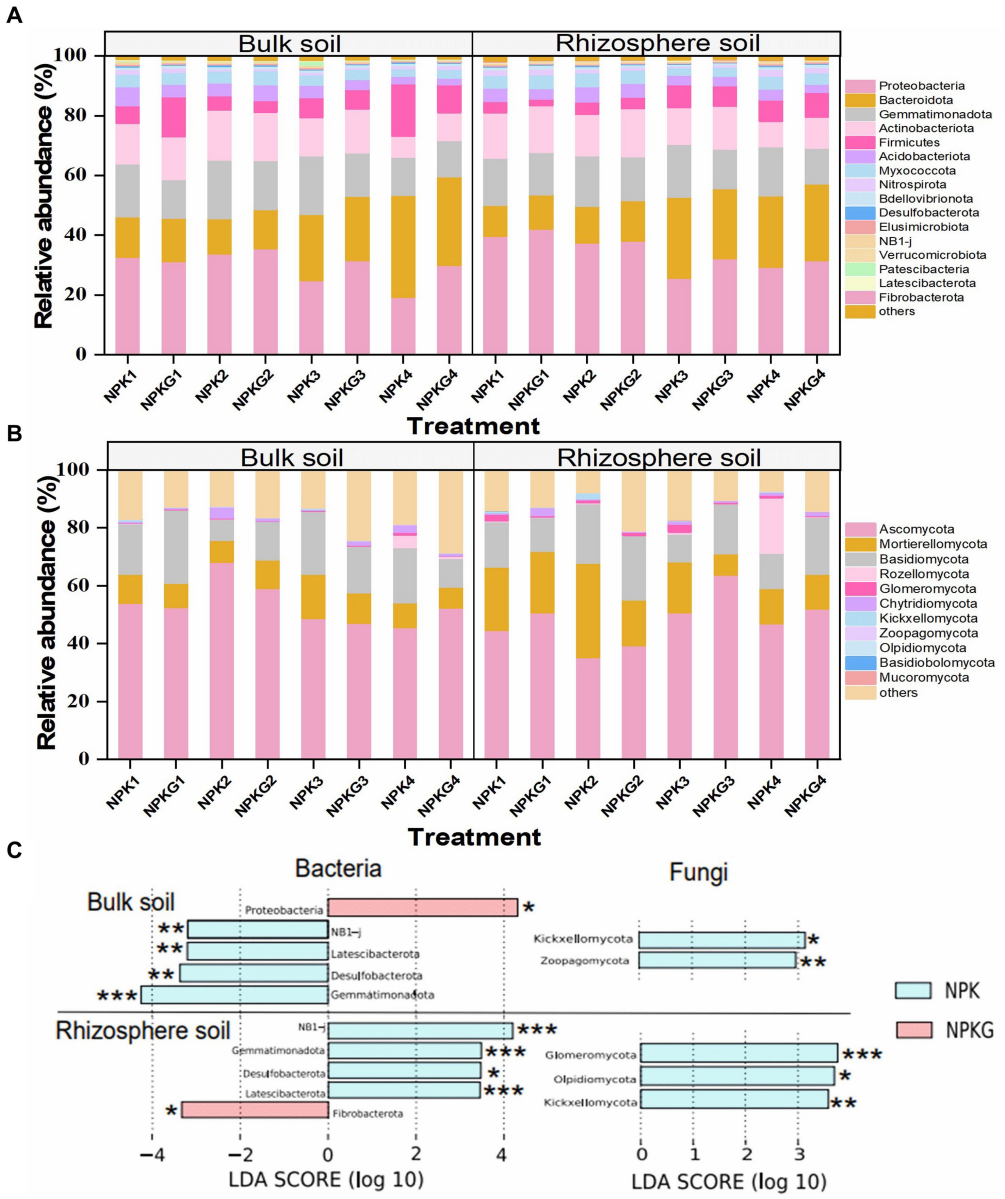
Relative abundance of the dominant **(A)** soil bacteria and **(B)** soil fungi at the phylum level. Biomarkers with statistical differences between treatment NPK and NPKG were determined by LEfSe **(C)**. * mean *P*<0.05, ** mean *P*<0.01, and *** mean *P*<0.001. NPK, no ryegrass, chemical fertilizer application only; NPKG, chemical fertilizer application with turning ryegrass. 1, 2, 3, and 4 in treatments represent 45 days and 75 days after transplanting in 2020, respectively, and 45 days and 75 days after transplanting in 2021.

The relative abundance and variance analyses of soil microbiome at the phylum level are shown in Figure 7. At the bacterial phylum level, the dominant bacteria (relative abundance >1%) in the bulk and rhizosphere soils were Proteobacteria, Bacteroidota, Gemmatimonadota, Actinobacteria, Firmicutes, Acidobacteria, Myxococcota, and Nitrospirota (Figure 7A). The LEfSe results showed that Gemmatimonadota, Latescibacterota, and Desulfobacterota were significantly reduced in the bulk and rhizosphere soils after ryegrass tillage and its return to the field. Bulk soil bacteria Proteobacteria and rhizosphere soil bacteria Fibrobacterota in the NPKG treatment were significantly higher than those in the NPK treatment group. At the fungal phylum level (Figure 7B), the fungi with an average relative abundance greater than 1% in bulk and rhizospheric soils were Ascomycota, Basidiomycota, Mortierellomycota, Rozellomycota, Glomeromycota, Chytridiomycota, Kickxellomycota, and Zoopagomycota. LEfSe results showed that the bulk soil fungi Zoopagomycota, Kickxellomycota, and rhizosphere soil Glomeromycota, Olpidiomycota, and Kickxellomycota were significantly reduced after ryegrass tillage and were returned to the field.

To further explore the changes in soil microbial communities, changes in soil microbial communities at the genus level were analyzed (Supplementary Figures S1, S2). At the bacterial genus level, the dominant bacterial genera shared by the bulk and rhizosphere soil was *Prevotella, Sphingomonas, AKAU4049, MND1, Muribaculaceae, S0134_terrestrial_group, Lysobacter, Nitrospira, Subgroup_7, Dongia*. The LEfSe results showed that tilling ryegrass significantly increased *Sphingomonas, Lysobacter,* and *Longimicrobiaceae* in bulk soil and *Sphingomonas* and *Pseudarthrobacter* in rhizosphere soil. The *AKAU4049* and *S0134_terrestrial_group* genera showed notable or extremely significant reductions in the bulk and rhizosphere soils.

At the fungal genus level, the dominant genera shared by the bulk and rhizosphere soils were *Mortierella, Chaetomium, Coprinellus, Neocosmospora, Minimedusa, Paurocotylis, Stachybotrys,* and *Preussia*. The soil fungi *Chaetomium, Minimedusa*, and *Paracylindrocarpon* increased significantly, whereas the bulk and rhizosphere soil fungi *Neocosmospora* markedly decreased after ryegrass tillage and its return to the field.

### 3.6. Correlation analysis between soil physicochemical properties and soil microbiome

**Figure 8:**
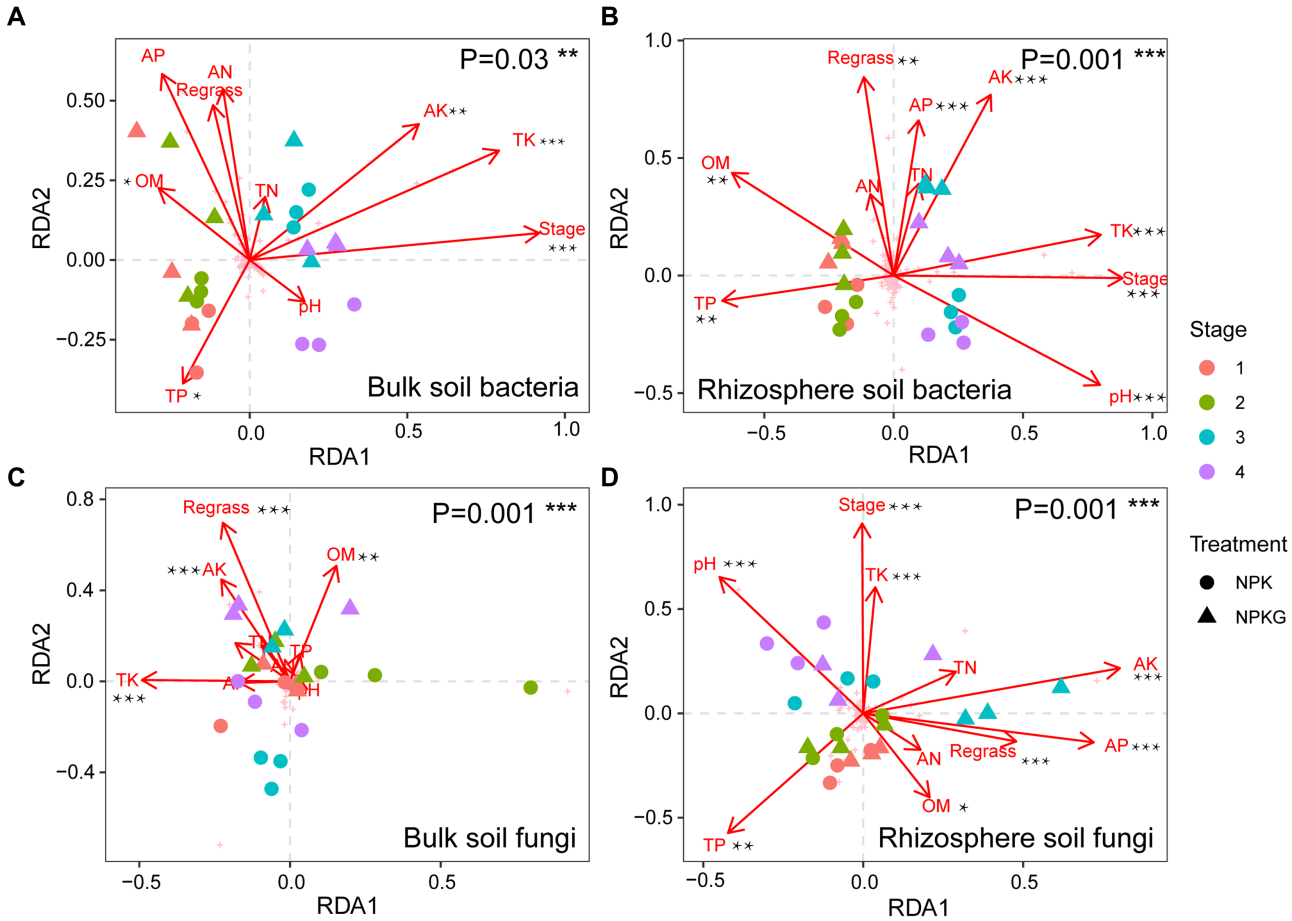
The correlation between microbial community structures bacteria **(A,B)** and fungi **(C,D)** and soil chemical properties based on RDA. NPK, no ryegrass, chemical fertilizer application only; NPKG, chemical fertilizer application with turning ryegrass. 1, 2, 3, and 4 in treatments represent 45 days and 75 days after transplanting in 2020, respectively, and 45 days and 75 days after transplanting in 2021. * mean *P*<0.05, ** mean *P*<0.01, and *** mean *P*<0.001.

**Figure 9:**
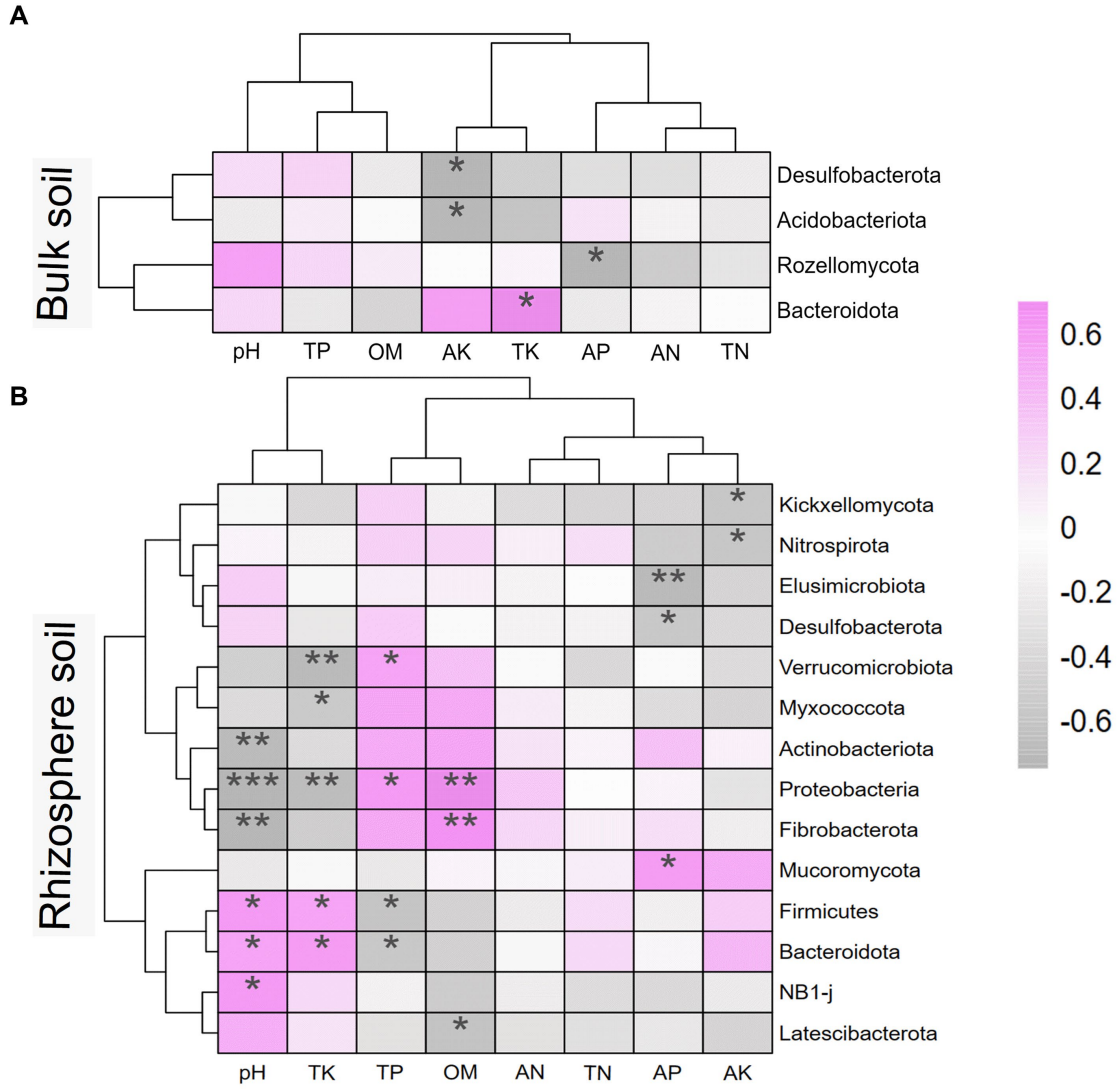
Heatmap of soil physicochemical properties and microbial communities at phylum level based on spearman correlation analysis. **(A)** is the bulk soil and **(B)** is the rhizosphere soil. * represents the correlation between soil microbiome and soil properties. * mean *P*<0.05, ** mean *P*<0.01, and *** mean *P*<0.001. AN, alkaline nitrogen; AP, available phosphorus; AK, available potassium; TN, total nitrogen; TP, total phosphorus; TK, total potassium; OM, organic matter.

To investigate the effects of soil environmental factors on the soil microbiome, RDA analyses were performed at the OTU level, and correlation heatmap analyses were conducted between the soil physicochemical properties and soil microbial community composition at the phylum level. As shown in Figure 8, the *p* values for all four models were less than 0.05, indicating that all soil environmental factors significantly affected soil microbiome. Figures 8A,C show that soil AK, TK, and OM significantly affected bulk soil bacterial and fungal communities. The rhizosphere soil AK, AP, and TK content, pH, and sampling period had significantly impacted on the rhizosphere soil microbial community. As shown in Figure 9, strongly correlations were observed between rhizosphere soil microbiome and rhizosphere soil nutrients. Rhizosphere soil pH showed strong positive correlations with the rhizosphere soil Firmicutes and Bacteroidota and highly significant negative correlations with the rhizosphere soil Proteobacteria, Actinobacteriota, and Fibrobacterota. Rhizosphere soil OM was significantly and positively correlated with the rhizosphere soil bacteria Proteobacteria and Fibrobacterota. Rhizosphere soil AK was significantly and adversely correlated with the rhizosphere soil fungi Kickxellomycota and bacteria Nitrospirota.

## 4. Discussion

### 4.1. Effects of returning ryegrass on soil layer construction and soil fertility

Generally, the soil profile is a comprehensive measure of the internal and external morphologies of soil. In this study, returning ryegrass to the field increased the thickness of the soil humus layer, and large soil particles were produced in the deposition layer. Returning ryegrass to the field also reduced the bulk density of soil humus and leaching layers. On the one hand, the well-developed and dense root system of ryegrass is deeply rooted in the soil to produce a drilling effect (Matocha et al., 2018; Pulido-Moncada et al., 2020), which reduces the soil density; on the other hand, the organic root exudates secreted by the roots of ryegrass dissolves the cohesives in the fragipan (Matocha et al., 2018).

The soil pH decreased after returning the ryegrass to the field, which was due to the ability of the ryegrass root system to secrete small molecule organic acids (Matocha et al., 2018) and the ryegrass itself released organic acids during dry decomposition to reduce the soil pH (Matocha et al., 2018). The soil nitrogen content increased after the ryegrass was returned to the field, which is consistent with the results of He et al. (2020). The green manure returned to the field is decomposed into soil nutrients and mineralized into inorganic nitrogen by the soil microbiome (Daly et al., 2021). The positive correlation between rhizospheric soil AN and bacteria Proteobacteria substantiated the important role of the microbiome in soil-available nutrients. Ryegrass tillage and return promote an increase in soil AP content, mainly because of the organic acids released by ryegrass roots to convert soil TP into the available state of phosphorus (Hansen et al., 2021; Yang X. Y. et al., 2021). The continuous return of ryegrass also significantly increased soil AK content, which was closely related to the soil type and ryegrass. The main components of yellow-brown soil are illite and vermiculite, which contain potassium minerals that plants cannot absorb or use. On the one hand, the ryegrass that has been turned and returned to the field can directly expand the interlayer space of clay minerals and release the interlayer mineral potassium (Rezaeinejad et al., 2021). Besides, ryegrass is degraded into humus by microorganisms, which can cause the expansion of clay minerals, thereby reducing the potassium fixation strength of the soil to exogenous potassium. OM is an important indicator of soil fertility. In this study, returning ryegrass to the field increased soil OM content, which is closely related to the positive priming effect of ryegrass on soil organic carbon mineralization (Li et al., 2018). The addition of ryegrass promoted the formation of larger pores in sandy loam soil and stimulated the mineralization of natural organic carbon (Mendoza et al., 2022), and the organic acids released by ryegrass could liberate OM by dissolving soil protective mineral phases (Ding et al., 2021).

### 4.2. Effects of returning ryegrass on agronomic characters and chemical constituents of flue-cured tobacco

The stem circumference, plant height, and dry matter weight of flue-cured tobacco were significantly higher after turning ryegrass, mainly because tilling ryegrass back into the field improved the soil structure and fertility. Ryegrass tillage increases the content of the rapidly AN, AP, AK, and OM in the soil, tillage of ryegrass reduces the soil bulk, loosens the soil, promotes root development, and enhances the ability of tobacco plants to absorb nutrients and water from the soil, thus facilitating the development and reproduction of tobacco plants. The content and coordination of the conventional chemical components in flue-cured tobacco leaves are important for their quality of tobacco leaves. Total sugar, reducing sugar, nicotine, chlorine, and potassium contents in tobacco measure the intrinsic quality, aroma, and taste of tobacco. In this study, ryegrass tillage increased the potassium content, which was closely related to the increase in soil AK content. Potassium in flue-cured tobacco is mainly derived from the soil system and is then absorbed into the body through the root system. The ratio of reducing sugar to nicotine is an important indicator for controlling the quality of cigarettes, which not only reflects the balance between acidic and alkaline substances in tobacco but also characterizes the coordination of the chemical composition of tobacco. In this study, returning ryegrass reduced the nicotine content, increased the potassium, total sugar, and reducing sugar contents, and reduced the sugar-to-nicotine ratio of flue-cured tobacco, indicating that returning ryegrass promotes harmonization of the chemical composition of flue-cured tobacco.

### 4.3. Effects of returning ryegrass on soil microbial community

An increase in soil fertility is inseparable from the contribution of the soil microbiome. In this study, the dominant phylum Proteobacteria increased after returning ryegrass, which is closely related to the organic carbon and OM produced by the decomposition of ryegrass in the soil. The strong positive correlation between rhizosphere soil OM content and Proteobacteria after ryegrass tillage and return to the field confirmed the response of Proteobacteria to soil OM, which is consistent with the findings of Li et al. (2019). Fibrobacterota is a cellulose-degrading bacterium (Ban et al., 2021), and an increase in Fibrobacterota in the rhizosphere soil after returning ryegrass promotes its degradation in the soil. Gemmatimonadetes, Latescibacterota, Desulfobacterota, and Kickxellomycota were dramatically reduced in both the bulk and rhizosphere soils after the ryegrass was tilled and pressed back into the field. Desulfobacterota and Kickxellomycota are anaerobes (Liu J. B. et al., 2022; Chen et al., 2023), and Latescibacterota decomposes complex organic compounds mainly under anaerobic conditions (Liu X. H. et al., 2022). Desulfobacterota, Latescibacterota, and Kickxellomycota decreased after returning to the ryegrass, indicating good aeration conditions in the soil. The significant negative relationship between Kickxellomycota and soil AK also demonstrated an increased soil pore structure. Gemmatimonadota prefers dry soil environments and exists in nutrient-poor environments. The reduction in Gemmatimonadota (including *S0134_terrestrial_group* genus and *AKAU4049* genus) in the soil after returning ryegrass also showed that the soil entropy of the returning ryegrass was suitable and rich in nutrients. Both *Lysobacter* and *Sphingomonas* genera were significantly higher in bulk soil with ryegrass than in soil without ryegrass. *Lysobacter* produces a variety of extracellular enzymes and metabolites with antagonistic activities against various soil-borne diseases. *Sphingomonas* also has the ability to degrade a wide range of organic pollutants (Yang Y. et al., 2021). An increased abundance of degrading microbiome indicates a reduced risk of crop damage by pests. The fungal genera *Chaetomium* and *Minimedusa* increased significantly, whereas *Neocosmospore* decreased in both the rhizosphere and bulk soil after returning ryegrass. *Chaetomium* not only promotes the degradation of OM, such as cellulose, cellulose disaccharides, and lignin but also has biocontrol effects on various plant pathogens. *Minimedusa* also has been shown to be effective against fungal diseases and has a good control effect (Wachowska and Rychcik, 2023). *Neocosmospore* is pathogenic genera that cause rot in the stems, roots, and tubers of Solanaceae crops (Sandoval-Denis et al., 2019; Custódio and Pereira, 2023). These results indicate that returning ryegrass to the field promotes soil aeration, increases the reproduction of microbiome with antibacterial functions, and inhibits the propagation of pathogenic microbiome.

## 5. Conclusion

Ryegrass tilling and returning to the field provides an important carbon source for soil microbiome and promotes microbiome reproduction. The large number of microbiome in turn promoted the decomposition of ryegrass, increased the thickness of the soil humus layer, and reduced the soil bulk density of the plow layer. Returning ryegrass into the field also provides a favorable environment for the reproduction of microbiome with antibacterial function that inhibits the propagation of pathogenic microorganisms and enhances the soil microorganism community toward the direction of making good use of soil nutrients, activating soil nutrients while establishing a new dynamic balance of soil nutrients, thereby improving the quality of cultivated land and raising the quality of flue-cured tobacco. This study provides a technical basis for using the winter idle time of continuous tobacco fields to plant and return ryegrass on limited arable land to alleviate the strain on arable land resources and improve tobacco yield and quality.

## Data availability statement

The data presented in the study are deposited in the NCBI repository, under accession numbers PRJNA990362 and PRJNA994851.

## Author contributions

HZ: Formal analysis, Writing – original draft. MZ: Investigation, Writing – original draft. JY: Data curation, Writing – review & editing. JW: Writing – review & editing. YC: Writing – review & editing. XY: Funding acquisition, Writing – review & editing, Conceptualization.
