# Supplementary material for: Returning ryegrass to continuous cropping soil improves soil nutrients and soil microbiome, producing good-quality flue-cured tobacco
Source: Front Microbiol. 2023 Oct 9;14:1257924. doi: 10.3389/fmicb.2023.1257924 (PMC10591219; doi:10.3389/fmicb.2023.1257924)
Supplement: Supplementary file 1 [file Data_Sheet_1.docx]

Supplementary Material

Returning ryegrass to continuous cropping soil improves soil nutrients and soil microbiome, producing good-quality flue-cured tobacco

**Hanjun Zhou ^1, 2, †^, Mingjie Zhang 1^, 3, †^, Jiahao Yang ^1^, Jing Wang ^1^, Yulu Chen ^1^, Xiefeng Ye ^1^, ***

***Corresponding Author:** Corresponding Author: [yexiefeng@henau.edu.cn](mailto:yexiefeng@henau.edu.cn)

† These authors contributed equally to this work.

# 1 Supplementary Figures





**Supplementary Figure 1.** Relative abundance of the dominant soil bacteria at the genus level of bulk (A) and rhizosphere (C) soil. Biomarkers with statistical differences between treatment NPK and NPKG were determined by LEfSe of bulk (B) and rhizosphere (D) soil, respectively. * mean P＜0.05, ** mean P＜0.01, and *** mean P＜0.001. NPK, no ryegrass, chemical fertilizer application only; NPKG, chemical fertilizer application with turning ryegrass. NPKB, and NPKGB represent the bulk soil of the treatment NPK and NPKG, respectively; NPKR, and NPKGR represent the rhizosphere soil of the treatment NPK and NPKG, respectively. 1, 2, 3, and 4 in treatments represent 45 d and 75 d after transplanting in 2020, respectively, and 45 d and 75 d after transplanting in 2021.





**Supplementary Figure 2.** Relative abundance of the dominant soil fungi at the genus level of bulk (A) and rhizosphere (C) soil. Biomarkers with statistical differences between treatment NPK and NPKG were determined by LEfSe of bulk (B) and rhizosphere (D) soil, respectively. * mean P＜0.05, ** mean P＜0.01, and *** mean P＜0.001. NPK, no ryegrass, chemical fertilizer application only; NPKG, chemical fertilizer application with turning ryegrass. NPKB, and NPKGB represent the bulk soil of the treatment NPK and NPKG, respectively; NPKR, and NPKGR represent the rhizosphere soil of the treatment NPK and NPKG, respectively. 1, 2, 3, and 4 in treatments represent 45 d and 75 d after transplanting in 2020, respectively, and 45 d and 75 d after transplanting in 2021.

# 2 Supplementary Tables

**Supplementary Table1.** Soil microbial sequence number

|  |  |  | Bacteria | | | Fungi | | |
| --- | --- | --- | --- | --- | --- | --- | --- | --- |
|  |  |  | valid_tags | OTUs | goods  coverage（%） | valid_tags | OTUs | goods  coverage（%） |
| 2020-45d | Bulk soil | NPK | 71566.3±1510.6 a | 5162.3±124.6 a | 0.979±0.00 a | 55602.5±643.0 a | 488.3±75.7 a | 0.998±0.00 a |
|  |  | NPKG | 67650.5±5766.3 a | 4776.0±897.7 a | 0.977±0.00 a | 38028.3±9005.5 b | 494.8±45.4 a | 0.997±0.00 ab |
|  | Rhizosphere soil | NPK | 72039.3±530.5 a | 4661.5±148.7 a | 0.980±0.00 a | 56069.0±1509.6 a | 535.5±50.5 a | 0.997±0.00 a |
|  |  | NPKG | 72622.0±1490.1 a | 4644.5±134.9 a | 0.979±0.00 a | 45439.5±10204.9 ab | 579.3±44.3 a | 0.995±0.00 b |
| 2020-75d | Bulk soil | NPK | 72262.5±793.5 a | 4668.3±70.1 ab | 0.978±0.00 a | 46393.8±14254.6 a | 439.8±28.4 a | 0.997±0.00 a |
|  |  | NPKG | 70634.5±1545.7 ab | 5321.5±110.6 a | 0.975±0.00 a | 50602.8±9864.2 a | 502.8±41.2 a | 0.996±0.00 a |
|  | Rhizosphere soil | NPK | 69993±914.7 b | 5343.5±91.2 a | 0.976±0.00 a | 42934.5±11886.8 a | 540.5±149.4 a | 0.996±0.00 a |
|  |  | NPKG | 70556.5±856.3 ab | 5275.0±180.4 a | 0.977±0.00 a | 39709.5±11886.8 a | 450.0±45.7 a | 0.996±0.00 a |
| 2021-45d | Bulk soil | NPK | 68023.5±1047.0 a | 5788.5±79.0 ab | 0.966±0.00 bc | 54386.0±10161.2 a | 380.0±21.7 ab | 0.998±0.00 a |
|  |  | NPKG | 65557.8±4608.2 a | 5955.5±334.1 a | 0.964±0.00 c | 63687.8±2141.2 a | 459.8±33.3 a | 0.996±0.00 b |
|  | Rhizosphere soil | NPK | 67083.0±719.3 a | 5399.8±255.8 b | 0.972±0.00 a | 44981.5±8158.1 a | 368.8±42.2 b | 0.998±0.00 a |
|  |  | NPKG | 66473.8±2216.1 a | 5552.3±303.8 ab | 0.970±0.00 ab | 64928.8±1493.0 a | 434.8±62.1 ab | 0.996±0.00 b |
| 2021-75d | Bulk soil | NPK | 65998.5±6077.3a | 5290.3±423.5b | 0.971±0.00 a | 48254.5±17024.1 a | 368.3±26.6 a | 0.998±0.00 a |
|  |  | NPKG | 67595.5±2793.5a | 5236.8±326.7b | 0.970±0.00 a | 55828.3±13250.2 a | 408.5±34.5 a | 0.997±0.00 b |
|  | Rhizosphere soil | NPK | 68556.5±929.8a | 5948.8±117.1a | 0.968±0.00 a | 55211.8±9196.8 a | 405.5±27.2 a | 0.997±0.00 b |
|  |  | NPKG | 67623.25±3131.9a | 5700.3±219.6ab | 0.970±0.00 a | 55975.8±10254.5 a | 430.5±59.6 a | 0.997±0.00 b |

Different lowercase letters indicate significant differences between treatments NPK and NPKG (P<0.05). NPK, no ryegrass, chemical fertilizer application only; NPKG, chemical fertilizer application with turning ryegrass.

**Supplementary Table 2.** Bacterial and fungal diversity analysis

|  | Stage |  | Treatment | Chao1 | Shannon | Observed_species | Simpson |
| --- | --- | --- | --- | --- | --- | --- | --- |
| Bacteria | 2020-45d | Bulk soil | NPK | 5970.41±129.13 a C | 10.55±0.03 a A | 4862.85±108.50 a BCD | 0.998±0.000 a A |
|  |  |  | NPKG | 6045.97±322.34 a C | 9.63±1.58 a A | 4533.65±778.35 a CD | 0.988±0.017 a A |
|  |  | Rhizosphere soil | NPK | 5794.73±254.23 a C | 10.15±0.26 a A | 4472.05±141.24 a CD | 0.997±0.001 a A |
|  |  |  | NPKG | 5794.73±254.24 a C | 10.06±0.19 a A | 4442.35±148.33 a CD | 0.997±0.001 a A |
|  | 2020-75d | Bulk soil | NPK | 5658.32±58.40 b C | 10.23±0.23 a A | 4352.38±83.82 b D | 0.997±0.000 a A |
|  |  |  | NPKG | 6346.85±128.98 a BC | 10.43±0.16 a A | 4998.03±89.24 a ABCD | 0.997±0.001 a A |
|  |  | Rhizosphere soil | NPK | 6623.66±195.64 a ABC | 10.36±0.07 a A | 5164.83±81.10 a ABCD | 0.997±0.000 a A |
|  |  |  | NPKG | 6728.57±169.78 a ABC | 10.29±0.04 a A | 5090.00±172.73 a ABC | 0.997±0.000 a A |
|  | 2021-45d | Bulk soil | NPK | 7205.88±108.52 a AB | 9.87±0.12 a A | 5421.70±71.14 a AB | 0.994±0.001 a A |
|  |  |  | NPKG | 7577.78±598.03 a A | 10.02±0.26 a A | 5659.13±452.85 a AB | 0.995±0.002 a A |
|  |  | Rhizosphere soil | NPK | 6861.86±331.46 a ABC | 9.59±0.31 a A | 5257.73±268.01 a ABC | 0.991±0.004 a A |
|  |  |  | NPKG | 7247.49±548.07 a AB | 9.87±0.22 a A | 5429.75±354.31 a AB | 0.994±0.001 a A |
|  | 2021-75d | Bulk soil | NPK | 6469.11±551.58 b ABC | 9.55±0.23 a A | 5030.45±311.78 bc ABCD | 0.989±0.004 a A |
|  |  |  | NPKG | 6577.30±300.30 b ABC | 9.55±0.16 a A | 4936.10±350.68 c ABCD | 0.991±0.003 a A |
|  |  | Rhizosphere soil | NPK | 7281.24±188.62 a AB | 9.85±0.05 a A | 5750.70±100.48 a A | 0.993±0.001 a A |
|  |  |  | NPKG | 7392.53±143.60 a AB | 9.84±0.22 a A | 5546.38±288.47 ab A | 0.993±0.002 a A |
| Fungi | 2020-45d | Bulk soil | NPK | 484.42±74.58 b ABC | 6.00±0.69 a A | 442.35±74.93 a AB | 0.948±0.027 a A |
|  |  |  | NPKG | 519.94±31.63 ab ABC | 5.57±0.49 a A | 458.53±53.21 a AB | 0.931±0.026 a A |
|  |  | Rhizosphere soil | NPK | 535.78±44.94 ab ABC | 5.97±1.04 a A | 474.00±53.21 a AB | 0.938±0.064 a A |
|  |  |  | NPKG | 603.10±30.09 a A | 5.68±0.19 a A | 499.20±22.51 a A | 0.949±0.005 a A |
|  | 2020-75d | Bulk soil | NPK | 456.31±44.92 a ABC | 5.30±1.47 a A | 405.55±39.60 a AB | 0.859±0.200 a A |
|  |  |  | NPKG | 508.97±18.64 a ABC | 5.77±0.20 a A | 440.40±28.23 a AB | 0.953±0.011 a A |
|  |  | Rhizosphere soil | NPK | 466.04±44.56 a ABC | 5.62±1.01 a A | 488.98±142.91 a AB | 0.937±0.040 a A |
|  |  |  | NPKG | 476.97±31.61 a ABC | 5.35±0.26 a A | 403.75±17.59 a AB | 0.941±0.011 a A |
|  | 2021-45d | Bulk soil | NPK | 379.74±42.91 a C | 5.33±0.88 a A | 333.63±38.08 a B | 0.914±0.081 a A |
|  |  |  | NPKG | 450.45±33.30 a ABC | 5.42±0.27 a A | 377.70±36.33 a AB | 0.944±0.010 a A |
|  |  | Rhizosphere soil | NPK | 396.07±38.04 a BC | 5.12±0.89 a A | 337.20±34.88 a B | 0.902±0.073 a A |
|  |  |  | NPKG | 440.91±55.05 a BC | 4.15±1.06 a A | 332.50±56.76 a B | 0.808±0.137 a A |
|  | 2021-75d | Bulk soil | NPK | 376.17±27.66 a C | 5.70±0.85 a A | 342.63±27.06 a AB | 0.939±0.044 a A |
|  |  |  | NPKG | 413.15±37.26 a BC | 5.03±0.33 a A | 346.18±34.95 a AB | 0.923±0.019 a A |
|  |  | Rhizosphere soil | NPK | 405.14±34.36 a BC | 4.76±1.20 a A | 351.90±36.35 a AB | 0.851±0.166 a A |
|  |  |  | NPKG | 430.81±52.25 a BC | 5.39±0.42 a A | 361.38±37.69 a AB | 0.945±0.020 a A |

Different lowercase letters indicate significant differences in the diversity of bulk and rhizosphere soil microbial communities for each sampling period (P<0.05); Different uppercase letters indicate the significant differences in the diversity of bulk and rhizosphere soil microbial communities for the four sampling periods (P<0.05). NPK, no ryegrass, chemical fertilizer application only; NPKG, chemical fertilizer application with turning ryegrass.
